# Circulating Soluble Endoglin Levels in Pregnant Women in Cameroon and Malawi—Associations with Placental Malaria and Fetal Growth Restriction

**DOI:** 10.1371/journal.pone.0024985

**Published:** 2011-09-22

**Authors:** Karlee L. Silver, Andrea L. Conroy, Rose G. F. Leke, Robert J. I. Leke, Philomina Gwanmesia, Malcolm E. Molyneux, Diane Taylor Wallace, Stephen J. Rogerson, Kevin C. Kain

**Affiliations:** 1 Sandra A. Rotman Laboratories, McLaughlin-Rotman Centre for Global Health, University Health Network-Toronto General Hospital, University of Toronto, Toronto, Ontario, Canada; 2 Department of Laboratory Medicine and Pathobiology, University of Toronto, Toronto, Ontario, Canada; 3 Faculty of Medicine and Biomedical Research, University of Yaoundé, Yaoundé, Cameroon; 4 Malawi-Liverpool-Wellcome Trust Clinical Research Programme, Blantyre, Malawi; 5 College of Medicine, University of Malawi, Blantyre, Malawi; 6 School of Tropical Medicine, University of Liverpool, Liverpool, United Kingdom; 7 Department of Biology, Georgetown University, Washington, D.C., United States of America; 8 Department of Tropical Medicine, John A. Burns School of Medicine, University of Hawaii, Honolulu, Hawaii, United States of America; 9 Department of Medicine, University of Melbourne, Royal Melbourne Hospital, Parkville, Victoria, Australia; 10 Tropical Disease Unit, Division of Infectious Diseases, Department of Medicine, University of Toronto, Toronto, Ontario, Canada; Weill Cornell Medical College, United States of America

## Abstract

Placental infections with Plasmodium falciparum are associated with fetal growth restriction resulting in low birth weight (LBW). The mechanisms that mediate these effects have yet to be completely described; however, they are likely to involve inflammatory processes and dysregulation of angiogenesis. Soluble endoglin (sEng), a soluble receptor of transforming growth factor (TGF)-β previously associated with preeclampsia in pregnant women and with severe malaria in children, regulates the immune system and influences angiogenesis. We hypothesized that sEng may play a role in development of LBW associated with placental malaria (PM). Plasma levels of sEng were measured in women (i) followed prospectively throughout pregnancy in Cameroon (n = 52), and (ii) in a case-control study at delivery in Malawi (n = 479). The relationships between sEng levels and gravidity, peripheral and placental parasitemia, gestational age, and adverse outcomes of PM including maternal anemia and LBW were determined. In the longitudinal cohort from Cameroon, 28 of 52 women (54%) experienced at least one malaria infection during pregnancy. In Malawi we enrolled two aparasitemic gravidity-matched controls for every case with PM. sEng levels varied over the course of gestation and were significantly higher in early and late gestation as compared to delivery (P<0.006 and P<0.0001, respectively). Circulating sEng levels were higher in primigravidae than multigravidae from both Cameroon and Malawi, irrespective of malarial infection status (p<0.046 and p<0.001, respectively). Peripheral parasitemia in Cameroonian women and PM in Malawian women were each associated with elevated sEng levels following correction for gestational age and gravidity (p = 0.006 and p = 0.033, respectively). Increased sEng was also associated with the delivery of LBW infants in primigravid Malawian women (p = 0.017); the association was with fetal growth restriction (p = 0.003) but not pre-term delivery (p = 0.286). Increased circulating maternal sEng levels are associated with P. falciparum infection in pregnancy and with fetal growth restriction in primigravidae with PM.

## Introduction

Low birth weight (LBW) is a well documented effect of malaria infections in pregnancy [1], and increases the risk of death in the first two years of life [2], [3]. These effects are most highly associated with *Plasmodium falciparum* infections of the placenta referred to as placental malaria (PM) [4]. Malaria infection during pregnancy also increases the risk of moderate or severe maternal anemia, which is another risk factor leading to LBW [5]. Since over 85 million pregnant women are at risk of *P. falciparum* infection every year [6], there is a need to better understand the mediators of poor clinical outcomes associated with PM. Such information should lead to better diagnosis and treatment of mothers who are at risk of having LBW babies.

LBW resulting from maternal malaria is associated with placental inflammation. This includes monocyte infiltration of the placental intervillous space [7], [8] and secretion of cytokines and chemokines [9], [10], [11]. In addition, there is a growing body of evidence that dysregulated angiogenic factors also contribute to LBW in the context of PM [12], [13]. Inflammation and angiogenesis are not mutually exclusive processes, but have complementary functions in the control of infection and injury and the limitation of detrimental tissue neo-vascularization [14], [15].

Soluble endoglin (sEng) is involved in both regulation of immune function and angiogenesis. It exerts these effects by binding and interfering with the activity of transforming growth factor (TGF)-β, which is a regulatory cytokine with multiple isoforms (e.g., TGF-β1, TGF-β3) and pleiotropic effects [16]. Specifically, sEng has been shown to reduce TGF-β1 bioavailability to membrane-bound TGF-β receptors and co-receptor Eng [17]. Eng is expressed on the membranes of endothelial cells in adult and fetal vessels, activated monocytes and macrophages, and placental syncytiotrophoblast [18], [19], [20]. The syncytiotrophoblast is also a source of sEng [17]. By binding TGF-β, sEng may inhibit the regulatory effects of TGF-β on the immune system. sEng also prevents angiogenic processes such as capillary formation and vascular permeability, and inhibits nitric oxide-mediated vasodilation [17] – processes that could affect the placental vascularization required for efficient growth of the developing fetus.

Elevation of plasma sEng levels has been shown to precede the onset of preeclampsia [17], [21], a pathological pregnancy condition that also increases the risk of preterm birth, fetal growth restriction and LBW. Elevated concentrations of circulating sEng have been associated with increased severity of malaria infection in children in Gabon [22]. To date, no published studies have reported on the relationship between PM, LBW and sEng levels. The purpose of this study was to determine whether sEng levels are increased with malaria in pregnancy and PM, and whether the increases are associated with delivery of LBW infants. We measured maternal peripheral plasma levels of sEng in two study populations; women prospectively followed over the course of pregnancy in Cameroon, and women studied at the time of delivery in Malawi. We report that increased sEng levels are associated with malaria infection and fetal growth restriction in a gravidity-dependent manner.

## Methods

### Ethics Statement

The prospective study in Cameroon was approved by the Institutional Review Board, Georgetown University and the National Ethical Committee, Ministry of Public Health, Cameroon. The cross-sectional study in Malawi received ethical approval from The College of Medicine Research and Ethics Committee in Blantyre, Malawi. Written informed consent was obtained from each study participant. In Cameroon, if written informed consent was unable due to illiteracy, documented verbal consent was obtained (i.e., a third party (usually a friend of the participant) signed that the woman had consented). This consent approach was specifically approved by the relevant review bodies.

### Participants and specimens

During 2001–2004, a cohort of pregnant women was prospectively followed over the course of pregnancy in Yaoundé and Ngali II, Cameroon [23]. The transmission of *P. falciparum* in Yaoundé is low with ∼13 infectious bites/person/year [24]. Ngali II, which is 30 km north-east of Yaoundé, is hyperendemic for malaria with approximately 265 infectious bites per person per year [23]. Last menstrual period was used to calculate gestational age at enrolment. Peripheral plasma samples were collected; thin and thick blood smears were prepared and examined for the presence of *P. falciparum* asexual parasites at monthly visits (up to seven visits per woman) spanning all three trimesters of pregnancy. Women who were blood-smear positive for malaria were prescribed antimalarial treatment according to the Cameroon Ministry of Health's policy. Peripheral plasma samples, thin and thick blood smears, intervillous space (IVS) blood and placental impression smears were collected at delivery, and infant weight was recorded. Levels of sEng were measured in samples from all study participants who satisfied the following inclusion criteria: primigravidae (first pregnancy) or multigravidae (≥3 pregnancies) with live singleton birth, minimum of three peripheral plasma samples from different gestation points available for testing, and PM status determined at delivery. A total of 244 peripheral plasma samples from 52 women were tested for sEng. Participants infected with *Plasmodium* species other than *P. falciparum* were not included in this study.

To further evaluate the association of sEng levels with PM, and with poor clinical outcomes of PM, a second study population was also tested: Between 2001 and 2006, pregnant women who attended the Queen Elizabeth Central Hospital (QECH) were invited to participate in the study following delivery of a live singleton newborn. QECH is a referral hospital in urban Blantyre where malaria transmission is seasonal with an average of 1 infective bite per person per year [25]. Cases were defined by the presence of *P. falciparum* asexual parasites present in the placental blood, as assessed by smear. For each case, two controls that were equal in age (±2 years) and gravidity, and were negative for malaria parasites by both peripheral and placental smear, were enrolled. All gravidities were included in this study population (n = 479) with multigravidae being defined as more than one prior pregnancy. Peripheral EDTA plasma samples were collected and stored at −80°C until testing. Mononuclear cell infiltration was assessed by microscopy, as previously described [7]: 500 intervillous blood cells under oil immersion were counted and classified as uninfected erythrocytes, infected erythrocytes or leukocytes (subdivided by morphology into lymphocytes, polymorphs or monocyte-macrophages). Those with peripheral *P. falciparum* infection but no sign of placental infection (n = 21) were not included in the sEng analysis.

Poor clinical outcomes include LBW (<2500 g), anemia (<11 g Hb/dL), growth restriction (defined as small-for-gestational-age: <10^th^ centile for growth, after [26]), and preterm birth (<37 weeks gestation).

### Plasma sEng measurements

Maternal peripheral plasma sEng levels were measured by ELISA with a commercially available human sEng kit (R&D Systems, Minneapolis, MN). All samples were tested with the investigators blinded to group and outcome.

### Statistical analyses

Continuous variables are reported as median [interquartile range (IQR)] and analyzed by Mann-Whitney U test. Categorical data were analyzed by Pearson's chi-square test or Fisher's exact test, as appropriate. A mixed linear multivariate model was applied to analyze the prospective study, with gestational age, gravidity, presence of peripheral parasites, and placental malaria infection as fixed variables, and study participant as a random variable. End of gestation (delivery), primigravidae, absence of peripheral parasitemia and absence of placental malaria infection were set as reference points for the fixed variables, respectively. Two-factor effects of log-transformed data (normalized) were analyzed by 2-way ANOVA; between-group effects were analyzed by Bonferonni post-tests. Correlations between continuous variables were analyzed by Spearman's correlation or partial correlation on log-transformed data to control for the effect of additional variables. All P values are two-sided. Statistical analyses were performed using Prism 4.03 (GraphPad Software, La Jolla, CA) or SPSS (Chicago, IL).

## Results

### Characteristics of study populations

**Figure 1:**
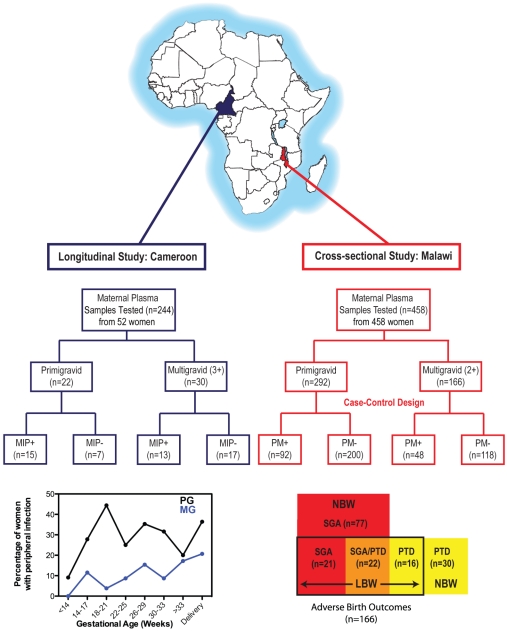
Overview of study populations from Cameroon and Malawi: timing of malaria infections during pregnancy in Cameroon and summary of adverse birth outcomes from Malawi. Women who participated in the longitudinal study in Cameroon were followed throughout gestation, at up to 7 antenatal visits and at delivery. Peripheral *P. falciparum* malaria infection was assessed by microscopy of peripheral blood smear at each visit; MIP+, blood-smear positive. Women who participated in the cross-sectional study in Malawi were enrolled at delivery. Placental malaria (PM) was determined by microscopy of placental blood smear. Adverse birth outcomes were defined as normal birth weight (NBW, ≥2500 g) or low birth weight (LBW, <2500 g) with or without growth restriction (small for gestational age (SGA), less than 10^th^ centile according to [26]) or preterm delivery (PTD, <37 weeks of gestation).

Characteristics of the prospective study population are listed in Table 1, and illustrated in Figure 1. Of 52 women who satisfied the criteria for the Cameroon study, 22 were primigravid and 30 were multigravid. As expected, multigravidae were older and delivered larger infants than primigravidae (Table 1). No difference was observed between gravidities in the gestational age of participants at enrolment, the number of antenatal visits, or the gestational age at delivery. Malaria infections were detected at least once (median [IQR] = 2 [1–3] times) in gestation (median [IQR] = 6 [5.5–7] prenatal visits) or at delivery in 28 women, 15 primigravidae and 13 multigravidae. 40% of the incidents of malaria infections were detected after 30 weeks of gestation, when the fetal growth velocity is at its highest [27]. The other 24 participants were blood-smear negative at all prenatal visits (median [IQR] = 6 [6–7] visits) and at delivery. Together, the primigravid women delivered infants with lower birth weight than multigravid women. Only 3 women (2 primigravid and 1 multigravid) delivered infants weighing <2500 g, this incidence of LBW being likely due to the effective provision of monthly prenatal care including malaria prophylaxis and iron supplementation [23].

**Table 1:** Characteristics of prospective study population (Cameroon).

	Primigravid (n = 22)	Multigravid (n = 30)	*P* value
**Age**, years	20 (18–24)	28 (24–32)	<0.0001
**Number** (%) **who were blood-smear positive ≥1 times during pregnancy**	15 (68%)	13 (43%)	0.096^a^
**Number** (%) **with PM at delivery**	7 (32%)	5 (17%)	0.320^a^
**Gestational age at enrolment**, weeks	14 (13–15.5)	13 (12–15.5)	0.201
**Number of antenatal visits**	6 (6–7)	6 (6–7)	0.289
**Gestational age at delivery**, weeks	39.5 (39–41)	40 (39–41)	0.831
**Birth weight**, g	2815 (2600–3200)	3400 (2975–3710)	0.0001

Data shown as median (interquartile range), except where noted.

a Fisher's exact test.

Characteristics of the cross-sectional study population are listed in Table 2, and illustrated in Figure 1. Since we enrolled two malaria-negative participants enrolled for every malaria-positive participant, one third of the women in each gravidity group were malaria positive. Parasites were detected in both the peripheral and placental compartments in 116 women (77 primigravidae and 39 multigravidae) and only placental parasites were detected in 24 women (15 primigravidae and 9 multigravidae). Malaria-infected primigravid women had on average higher levels of placental parasitemia (Table 2) and mononuclear cell infiltration (primigravid, 10.0 [0–22.5] vs. multigravid, 5.0 [0–18.8] mononuclear cells per 500 cells; p = 0.016) than infected multigravid women. Multigravidae were older and delivered infants with higher birth weight than primigravidae.

**Table 2:** Characteristics of cross-sectional study population (Malawi).

	Primigravid (n = 292)	Multigravid (n = 166)	*P* value
**Age**, years	18.0 (17.0–20.0)	25.0 (22.0–28.0)	<0.001
**Hemoglobin concentration**, g/dL	12.0 (10.5–13.3)	11.8 (10.6–12.9)	0.388
**Number** (%) **with PM at delivery** a	92 (31.5)	48 (28.9)	0.563
**Placental parasites in infected mothers**, per µL	70.0 (21.0–446.0)	40.0 (9.3–111.5)	0.013
**Gestational age at delivery**, weeks	38 (38–40)	40 (38–40)	<0.001
**Birth weight**, g	2800 (2563–3138)	3150 (2900–3450)	<0.001

Data shown as median (interquartile range), except where noted.

a Product of study design with two malaria-negative control enrolled for each malaria-positive case enrolled.

### Soluble endoglin levels change with gestation and gravidity

**Figure 2:**
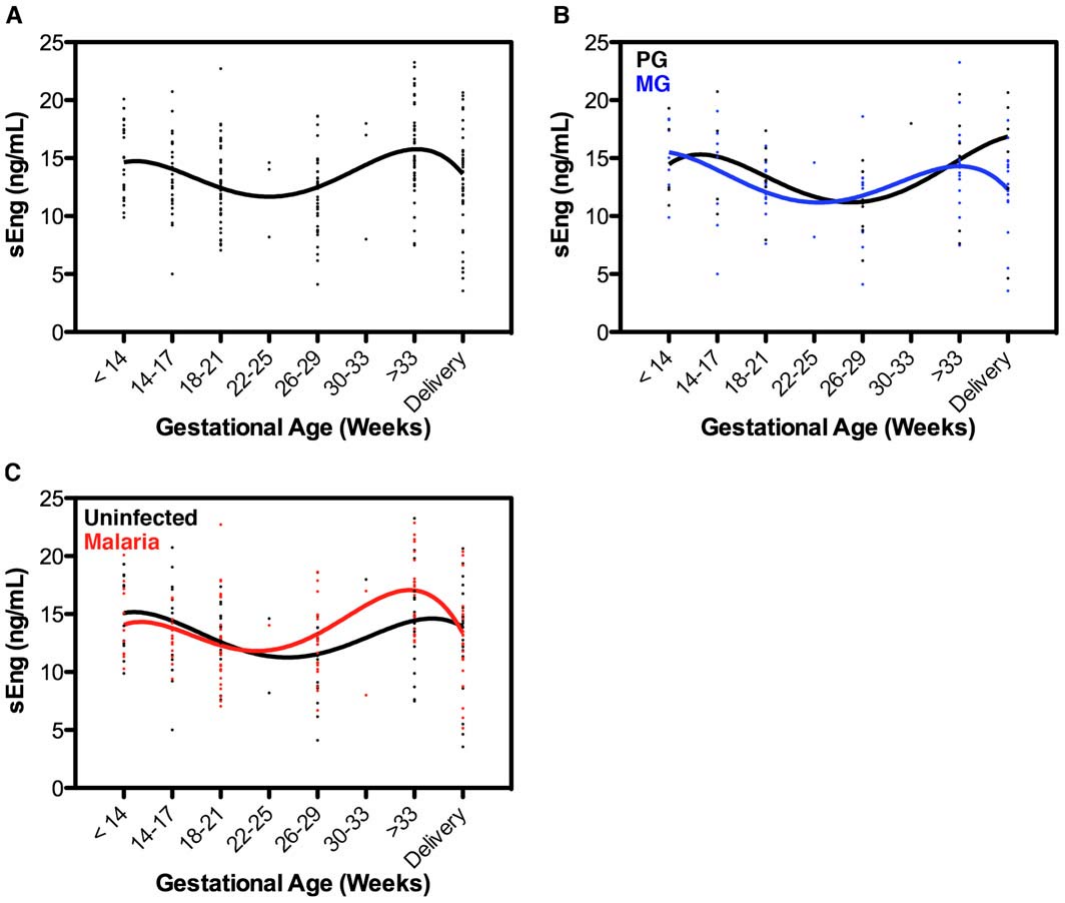
Maternal sEng levels are affected by gravidity, gestational age and malaria infection. sEng levels measured in peripheral plasma samples from study participants were peripheral blood-smear negative at all study visits, shown as *(A)* all gravidities together, and *(B)* split into primigravidae (PG) and multigravidae (MG). *(C)* Levels of sEng from all study participants split into those with *P. falciparum* infection detected at one or more antenatal visit or at delivery (Malaria) or blood-smear negative at all study visits (Uninfected). Multi-order polynomial non-linear regression curves with the best fits to the data are depicted to illustrate the effect trends in peripheral sEng levels throughout gestation.

Since sEng is secreted by the placental syncytiotrophoblast and has anti-angiogenic effects on endothelial cells that might be important for physiologic placental vascular remodelling during gestation [17], we first examined if its expression is altered with the stage of gestation in the prospective study population in Cameroon. Mean maternal peripheral sEng levels varied with gestation age (p<0.0001 by univariate analysis). In a multivariate analysis taking into account gestational age, gravidity and presence of peripheral malaria infection, mean sEng concentration was significantly higher in early (<14 weeks) and late (>33 weeks) gestation than at delivery (p<0.004 and p<0.0001, respectively; Fig. 2A).

### Soluble endoglin levels change with gravidity

Gravidity is an important factor in the susceptibility to PM, with primigravidae being more susceptible to both infection and to sequelae resulting from infection [4]. The same multivariate analysis on the Cameroonian study population results, accounting for gestational age and the presence of peripheral or placental parasites, showed that mean maternal sEng levels were higher in primigravid than in multigravid women (p<0.046; Fig. 2B). This association was also observed at delivery in the Malawian population, with primigravidae having higher median sEng levels (50.2 [39.5–64.3] ng/mL) than multigravidae (43.2 [31.9–52.8] ng/mL; p<0.001).

### Malaria in pregnancy increases soluble endoglin levels

**Figure 3:**
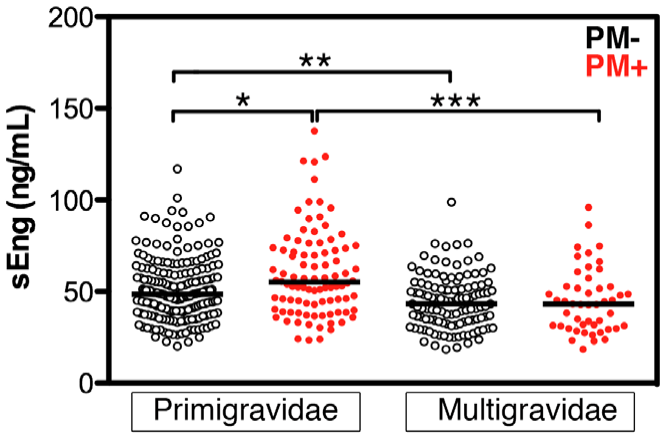
Maternal sEng at delivery is increased with placental malaria in primigravidae and not multigravidae. sEng was measured in peripheral plasma samples of Malawian women at delivery. Placental malaria (PM+) was defined as positive by placental blood smear microscopy. Bars represent medians. 2-way ANOVA on log-transformed data: gravidity, p<0.0001. *, p<0.05; **, p<0.01; ***, p<0.001 by Bonferonni post-test.

In the Cameroonian study population, the presence of peripheral parasitemia during pregnancy was associated with an increase in maternal sEng levels when gestational age and gravidity were taken into account (p = 0.006; Fig. 2C). Likewise, maternal sEng was increased with PM infection in the Malawian population (p<0.05), after controlling for gestational age and gravidity (Fig. 3).

Since the Malawian cross-sectional study population had a larger number of women, it was possible to further explore the association of increased sEng and PM at delivery. A weak but significant correlation was observed between peripheral sEng levels and peripheral or placental parasitemia (peripheral: Spearman's rho = 0.198, p = 0.001; placental: rho = 0.179, p = 0.002). Among women with placental malaria infection, sEng was positively correlated with placental monocyte infiltration (rho = 0.127, p = 0.007); however, the relationship was strongest in primigravidae with PM (rho = 0.303, p = 0.004).

### Soluble endoglin levels are highest among women with poor clinical outcomes of malaria in pregnancy

**Figure 4:**
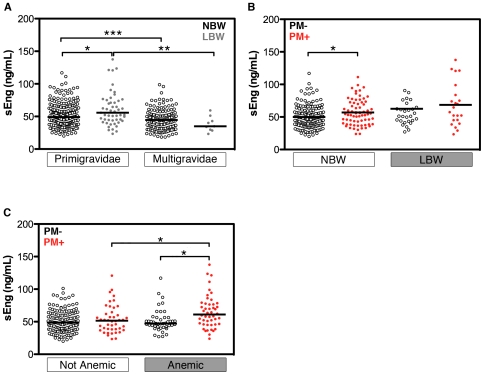
Elevated maternal sEng at delivery is associated with poor clinical outcomes in primigravidae. sEng was measured in peripheral plasma samples of Malawian women at delivery. Results displayed by (A) gravidity and birth weight; (B) birth weight and placental malaria (PM) status for primigravidae only; (C) anemia and PM status for primigravidae only. PM was defined as *P. falciparum* positivity in placental blood smear by microscopy. Anemia was defined as <11 g Hb/dL. NBW, normal birth weight; LBW, low birth weight. Bars represent medians. *, p<0.05; **, p<0.01; ***, p<0.001 by Bonferonni post-test of 2-way ANOVA on log-transformed data.

In the Malawian study population, 51 (17.5%) of the primigravid women and 9 (5.5%) of the multigravid women delivered LBW infants. Among primigravidae only, the median maternal sEng level was higher in mothers of LBW infants than in mothers of normal birth weight infants (Mann-Whitney, p = 0.017; Fig. 4A). These higher concentrations of sEng in primigravidae were associated with fetal growth restriction (small for gestational age (n = 92) vs normal for gestational age (n = 197), p = 0.003) but not with preterm birth (preterm (n = 54) vs term birth (n = 235), p = 0.286).

Stratification of primigravid participants by malaria infection and birth weight illustrates that both PM and LBW outcomes were associated with increased peripheral sEng levels at delivery (2-way ANOVA (log_10_[sEng]): PM, p = 0.008; LBW, p = 0.006; Fig. 4B). Peripheral sEng levels in primigravidae with malaria were negatively correlated with birth weight when correcting for gestational age at delivery (rho = −0.237, p<0.001). This correlation was not observed in the multigravid group (rho = 0.031, p = 0.693).

We also examined the change in sEng with anemia, another poor clinical outcome associated with both PM and LBW [5]. Anemia was associated with an increase in sEng in primigravidae (p = 0.019; Fig. 4C). However, in a multivariable logistic regression model with age and parasitemia as covariates, sEng was not an independent predictor of anemia (p = 0.102).

## Discussion

Circulating sEng levels correlate with severity of disease in both preeclamptic women and *P. falciparum*-infected children [17], [22]. By measuring sEng in maternal plasma at various stages of pregnancy in a longitudinal study in Cameroon and in a large case-control cross-sectional study from Malawi, we were able to show that circulating sEng levels were associated with poor outcomes of malaria infection in pregnancy. sEng was increased in pregnant women with peripheral malaria parasitemia during pregnancy, and peripheral sEng levels at delivery were increased in the presence of PM. Maternal sEng levels at delivery also correlated with fetal growth restriction.

These two geographically distinct populations showed similar increases in sEng levels in primigravidae and malaria infected women, despite differences in malaria endemicity and likely diversity in host and parasite genetics. These results suggest that these findings may be generalizable to other populations.

The source of the circulating sEng we measured in maternal plasma samples is unknown. Cell types that have been implicated in PM, including endothelial cells, activated monocytes and macrophages, have been shown to express Eng [18], [19], [20]. Syncytiotrophoblast is a source of sEng [17]; however sEng has been detected in non-pregnant individuals (e.g., children with severe malaria [22]), and thus is likely also cleaved from the surface of other Eng-expressing cells by matrix metalloproteinases [28].

The changes we observed in the levels of sEng with malaria were small, though consistent. This small range coupled with natural variation between individuals pre-empts the use of sEng as a stand-alone biomarker for either PM or for individuals at risk of poor outcomes. However, it may prove to be more informative when coupled with other biomarkers of PM, as is the case for preeclampsia, where the utility of sEng as a prognostic biomarker is strengthened when combined with measurements of soluble Flt-1 and placental growth factor [21], [29].

Our findings, coupled with an understanding of the biological effects of sEng, lead us to suggest that sEng may play a role in mediating the pathogenesis of fetal growth restriction associated with PM. sEng binds to TGF-β, and thereby limits TGF-β bioavailability [17]. The increase of sEng we observed in maternal plasma of primigravid women with PM is consistent with reports that PM is associated with a decrease in maternal and placental TGF-β levels [10], [30], [31]. The levels of TGF-β1 have also been shown to be inversely correlated with malaria disease severity [32], [33], and to be important in dictating severity of pathology in murine models of malaria [34], [35], [36]. TGF-β has multiple biological functions, including immune regulation, vascular development, and hematopoeisis [37], [38], [39]. sEng may be mediating LBW in the context of PM by interfering with any of these effects, and, owing to the pleiotropic nature of its ligand, it is likely that any effect sEng may have on fetal growth restriction occurs through several mechanisms.

Monocytic infiltration of the placenta is a strong correlate of LBW in PM [7], [8]. TGF-β can have both pro- and anti-inflammatory effects on immune cells from the monocyte/macrophage lineage depending on the activation state of the cells [16]. TGF-β is a potent chemoattractant for monocytes and has been shown to potentiate inflammation via IL-1 and IL-6 secretion [40], [41], [42]. TGF-β also induces monocyte secretion of matrix metalloproteinases [41]. In this way sEng may be cleaved from syncytiotrophoblast to control TGF-β–dependent monocyte infiltration and inflammation. A model whereby sEng is cleaved in response to monocyte infiltration is supported by the correlation between mononuclear cell infiltrates and sEng levels in Malawian primigravidae with PM.

Once monocytes differentiate into macrophages, TGF-β induces an anti-inflammatory effect by inhibiting secretion of TNF, chemokines, and nitric oxide [16]. This effect was observed in mice in whom recombinant TGF-β treatment induced IL-10, decreased TNF, and prolonged survival time to infection with the rodent parasite *Plasmodium berghei* [35]. Conversely, increases in sEng that result in reduction of bioactive TGF-β in PM may interfere with this anti-inflammatory role, and favour the establishment of a pro-inflammatory environment. A ratio of TNF∶IL-10 that favours TNF has been associated with severe malaria in children [33], and with LBW outcomes of PM [11].

Increased sEng may also mediate fetal growth restriction by acting directly on the placental vasculature. This contention is supported by our observations that circulating sEng is expressed at higher amounts at times in gestation that correspond to uterine invasion by trophoblast (early gestation) [43] and active vascular remodelling to support optimal fetal growth (last trimester; Fig. 2A). TGF-β is known to both inhibit trophoblast proliferation, migration and uterine invasion [44], [45], and to promote endothelial cell proliferation and migration [46], depending on the signalling pathway and cell type targeted. sEng has been shown to reduce endothelial cell sprouting and capillary vasodilation by blocking TGF-β activity [17]. Increased levels of sEng have been correlated with increased impedance to uterine and umbilical arterial blood flow [47]. The early increase in sEng level, which does not appear to change with gravidity (Fig. 2A), is likely required for normal placentation while malaria-induced increases in sEng in later gestation (Fig. 2C) may restrict TGF-β–mediated placental vessel growth and lead to functional placental insufficiency and impaired fetal growth.

A third way that sEng may mediate fetal growth restriction is by dysregulating erythropoeisis. We observed an increase in sEng levels with maternal anemia in primigravidae, and previous reports have associated anemia with LBW [11], [48]. Erythrocyte proliferation and differentiation requires TGF-β signalling through membrane-bound Eng [39], [49], thus decreased bioavailable TGF-β as a result of increased sEng may reduce the number of erythrocytes in circulation and, therefore, the amount of oxygen that is delivered to the fetus. Given that sEng was not an independent predictor of anemia, it is also possible that high parasitemia caused proportional but unrelated variations in both sEng levels and hemoglobin levels in primigravidae.

The mechanism(s) by which sEng may mediate growth restriction in PM will be more thoroughly informed by a prospective study that involves serial blood sampling together with ultrasound measurements of fetal growth to assess how changes in sEng at different points in gestation related to fetal development. It would also be informative to measure TGF-β alongside sEng; however, circulating TGF-β must be measured in platelet poor plasma, which was not collected from the current study populations.

HIV infection status was unknown in our study participants, but the prevalence of HIV infection in pregnant women was 7% and 30% in Cameroon and Malawi, respectively, during the periods of recruitment [50], [51]. While HIV, and other co-infections, may affect sEng levels, it is unlikely to be a significant confounder of the results presented in this study, as the two populations with different levels of HIV prevalence showed the same results.

To our knowledge, this is also the first study to describe elevated sEng levels in first pregnancies. Primigravidae are known to be more susceptible to malaria infection and sequelae associated with malaria [52]. The increased susceptibility in primigravidae is thought to be primarily due to a lack of antibodies specific for placental chondroitin sulphate A (CSA)-binding parasites that can prevent sequestration and promote opsonic clearance of infected erythrocytes in the placenta [11], [53], [54]. Primigravidae are also at higher risk of developing preeclampsia. Immune maladaption to the semi-allogenic fetus has been suggested as an explanation for this susceptibility [55]. Higher baseline systemic levels of sFlt-1, an anti-inflammatory and anti-angiogenic protein, have also been noted in first time pregnancies [56]. Increased constitutive sFlt-1 and sEng expression may keep heightened basal immune system activation in check, but also predispose primigravidae to poor clinical outcomes such as hypertension and fetal growth restriction. The absence of increased sEng in malaria-infected multigravidae (Fig. 2 and 4A), i.e., those less susceptible to PM sequelae, is also consistent with this hypothesis.

In conclusion, we found that maternal circulating sEng is increased with *P. falciparum* infection in pregnancy and with fetal growth restriction in primigravidae with PM. These changes are not sufficient for sEng on its own to be a useful biomarker of these conditions, but its potential in combination with other biomarkers deserves further study. Our findings are compatible with the hypothesis that sEng may play a role in the pathogenesis of PM-related fetal growth restriction.

## References

[1] Guyatt HL, Snow RW (2004). Impact of malaria during pregnancy on low birth weight in sub-Saharan Africa.. Clin Microbiol Rev.

[2] Guyatt HL, Snow RW (2001). Malaria in pregnancy as an indirect cause of infant mortality in sub-Saharan Africa.. Trans R Soc Trop Med Hyg.

[3] MacDorman MF, Atkinson JO (1999). Infant mortality statistics from the 1997 period linked birth/infant death data set.. Natl Vital Stat Rep.

[4] McGregor IA, Wilson ME, Billewicz WZ (1983). Malaria infection of the placenta in The Gambia, West Africa; its incidence and relationship to stillbirth, birthweight and placental weight.. Trans R Soc Trop Med Hyg.

[5] Steketee RW, Nahlen BL, Parise ME, Menendez C (2001). The burden of malaria in pregnancy in malaria-endemic areas.. Am J Trop Med Hyg.

[6] Dellicour S, Tatem AJ, Guerra CA, Snow RW, ter Kuile FO (2010). Quantifying the number of pregnancies at risk of malaria in 2007: a demographic study.. PLoS Med.

[7] Rogerson SJ, Pollina E, Getachew A, Tadesse E, Lema VM (2003). Placental monocyte infiltrates in response to Plasmodium falciparum malaria infection and their association with adverse pregnancy outcomes.. Am J Trop Med Hyg.

[8] Menendez C, Ordi J, Ismail MR, Ventura PJ, Aponte JJ (2000). The impact of placental malaria on gestational age and birth weight.. J Infect Dis.

[9] Rogerson SJ, Brown HC, Pollina E, Abrams ET, Tadesse E (2003). Placental tumor necrosis factor alpha but not gamma interferon is associated with placental malaria and low birth weight in Malawian women.. Infect Immun.

[10] Moormann AM, Sullivan AD, Rochford RA, Chensue SW, Bock PJ (1999). Malaria and pregnancy: placental cytokine expression and its relationship to intrauterine growth retardation.. J Infect Dis.

[11] Fried M, Muga RO, Misore AO, Duffy PE (1998). Malaria elicits type 1 cytokines in the human placenta: IFN-gamma and TNF-alpha associated with pregnancy outcomes.. J Immunol.

[12] Silver KL, Zhong K, Leke RG, Taylor DW, Kain KC (2010). Dysregulation of angiopoietins is associated with placental malaria and low birth weight.. PLoS One.

[13] Muehlenbachs A, Mutabingwa TK, Edmonds S, Fried M, Duffy PE (2006). Hypertension and maternal-fetal conflict during placental malaria.. PLoS Med.

[14] Fiedler U, Augustin HG (2006). Angiopoietins: a link between angiogenesis and inflammation.. Trends Immunol.

[15] Pober JS, Sessa WC (2007). Evolving functions of endothelial cells in inflammation.. Nat Rev Immunol.

[16] Li MO, Wan YY, Sanjabi S, Robertson AK, Flavell RA (2006). Transforming growth factor-beta regulation of immune responses.. Annu Rev Immunol.

[17] Venkatesha S, Toporsian M, Lam C, Hanai J, Mammoto T (2006). Soluble endoglin contributes to the pathogenesis of preeclampsia.. Nat Med.

[18] Gougos A, Letarte M (1988). Identification of a human endothelial cell antigen with monoclonal antibody 44G4 produced against a pre-B leukemic cell line.. J Immunol.

[19] Gougos A, St Jacques S, Greaves A, O'Connell PJ, d'Apice AJ (1992). Identification of distinct epitopes of endoglin, an RGD-containing glycoprotein of endothelial cells, leukemic cells, and syncytiotrophoblasts.. Int Immunol.

[20] Lastres P, Bellon T, Cabanas C, Sanchez-Madrid F, Acevedo A (1992). Regulated expression on human macrophages of endoglin, an Arg-Gly-Asp-containing surface antigen.. Eur J Immunol.

[21] Levine RJ, Lam C, Qian C, Yu KF, Maynard SE (2006). Soluble endoglin and other circulating antiangiogenic factors in preeclampsia.. N Engl J Med.

[22] Dietmann A, Helbok R, Lackner P, Fischer M, Reindl M (2009). Endoglin in African children with Plasmodium falciparum malaria: a novel player in severe malaria pathogenesis?. J Infect Dis.

[23] Leke RF, Bioga JD, Zhou J, Fouda GG, Leke RJ (2010). Longitudinal studies of Plasmodium falciparum malaria in pregnant women living in a rural Cameroonian village with high perennial transmission.. Am J Trop Med Hyg.

[24] Manga L, Robert V, Mess J, Desfontaine M, Carnevale P (1992). Le paludisme urbain a Yaounde, Cameroon: l'etude entomologique dans deux quartiers centraux.. Mem Soc R Belge Entomol.

[25] Calis JC, Phiri KS, Faragher EB, Brabin BJ, Bates I (2008). Severe anemia in Malawian children.. N Engl J Med.

[26] Landis SH, Ananth CV, Lokomba V, Hartmann KE, Thorp JM (2009). Ultrasound-derived fetal size nomogram for a sub-Saharan African population: a longitudinal study.. Ultrasound Obstet Gynecol.

[27] Rogerson SJ, Mwapasa V, Meshnick SR (2007). Malaria in pregnancy: linking immunity and pathogenesis to prevention.. Am J Trop Med Hyg.

[28] Velasco-Loyden G, Arribas J, Lopez-Casillas F (2004). The shedding of betaglycan is regulated by pervanadate and mediated by membrane type matrix metalloprotease-1.. J Biol Chem.

[29] Romero R, Nien JK, Espinoza J, Todem D, Fu W (2008). A longitudinal study of angiogenic (placental growth factor) and anti-angiogenic (soluble endoglin and soluble vascular endothelial growth factor receptor-1) factors in normal pregnancy and patients destined to develop preeclampsia and deliver a small for gestational age neonate.. J Matern Fetal Neonatal Med.

[30] Abrams ET, Kwiek JJ, Mwapasa V, Kamwendo DD, Tadesse E (2005). Malaria during pregnancy and foetal haematological status in Blantyre, Malawi.. Malar J.

[31] Abrams ET, Milner DA, Kwiek J, Mwapasa V, Kamwendo DD (2004). Risk factors and mechanisms of preterm delivery in Malawi.. Am J Reprod Immunol.

[32] Chaiyaroj SC, Rutta AS, Muenthaisong K, Watkins P, Na Ubol M (2004). Reduced levels of transforming growth factor-beta1, interleukin-12 and increased migration inhibitory factor are associated with severe malaria.. Acta Trop.

[33] Perkins DJ, Weinberg JB, Kremsner PG (2000). Reduced interleukin-12 and transforming growth factor-beta1 in severe childhood malaria: relationship of cytokine balance with disease severity.. J Infect Dis.

[34] Omer FM, de Souza JB, Riley EM (2003). Differential induction of TGF-beta regulates proinflammatory cytokine production and determines the outcome of lethal and nonlethal Plasmodium yoelii infections.. J Immunol.

[35] Omer FM, Riley EM (1998). Transforming growth factor beta production is inversely correlated with severity of murine malaria infection.. J Exp Med.

[36] Li C, Sanni LA, Omer F, Riley E, Langhorne J (2003). Pathology of Plasmodium chabaudi chabaudi infection and mortality in interleukin-10-deficient mice are ameliorated by anti-tumor necrosis factor alpha and exacerbated by anti-transforming growth factor beta antibodies.. Infect Immun.

[37] Letterio JJ, Roberts AB (1998). Regulation of immune responses by TGF-beta.. Annu Rev Immunol.

[38] Rossant J, Howard L (2002). Signaling pathways in vascular development.. Annu Rev Cell Dev Biol.

[39] Cho SK, Bourdeau A, Letarte M, Zuniga-Pflucker JC (2001). Expression and function of CD105 during the onset of hematopoiesis from Flk1(+) precursors.. Blood.

[40] Wiseman DM, Polverini PJ, Kamp DW, Leibovich SJ (1988). Transforming growth factor-beta (TGF beta) is chemotactic for human monocytes and induces their expression of angiogenic activity.. Biochem Biophys Res Commun.

[41] Wahl SM, Hunt DA, Wakefield LM, McCartney-Francis N, Wahl LM (1987). Transforming growth factor type beta induces monocyte chemotaxis and growth factor production.. Proc Natl Acad Sci U S A.

[42] Turner M, Chantry D, Feldmann M (1990). Transforming growth factor beta induces the production of interleukin 6 by human peripheral blood mononuclear cells.. Cytokine.

[43] Pijnenborg R, Bland JM, Robertson WB, Dixon G, Brosens I (1981). The pattern of interstitial trophoblastic invasion of the myometrium in early human pregnancy.. Placenta.

[44] Graham CH, Lala PK (1991). Mechanism of control of trophoblast invasion in situ.. J Cell Physiol.

[45] Caniggia I, Taylor CV, Ritchie JW, Lye SJ, Letarte M (1997). Endoglin regulates trophoblast differentiation along the invasive pathway in human placental villous explants.. Endocrinology.

[46] Goumans MJ, Valdimarsdottir G, Itoh S, Rosendahl A, Sideras P (2002). Balancing the activation state of the endothelium via two distinct TGF-beta type I receptors.. EMBO J.

[47] Chaiworapongsa T, Romero R, Kusanovic JP, Mittal P, Kim SK (2010). Plasma soluble endoglin concentration in pre-eclampsia is associated with an increased impedance to flow in the maternal and fetal circulations.. Ultrasound Obstet Gynecol.

[48] Kidanto HL, Mogren I, Lindmark G, Massawe S, Nystrom L (2009). Risks for preterm delivery and low birth weight are independently increased by severity of maternal anaemia.. S Afr Med J.

[49] Moody JL, Singbrant S, Karlsson G, Blank U, Aspling M (2007). Endoglin is not critical for hematopoietic stem cell engraftment and reconstitution but regulates adult erythroid development.. Stem Cells.

[50] Kongnyuy EJ, Soskolne V, Adler B (2008). Hormonal contraception, sexual behaviour and HIV prevalence among women in Cameroon.. BMC Womens Health.

[51] Kwiek JJ, Mwapasa V, Alker AP, Muula AS, Misiri HE (2008). Socio-demographic characteristics associated with HIV and syphilis seroreactivity among pregnant women in Blantyre, Malawi, 2000–2004.. Malawi Med J.

[52] Duffy PE (2007). Plasmodium in the placenta: parasites, parity, protection, prevention and possibly preeclampsia.. Parasitology.

[53] Keen J, Serghides L, Ayi K, Patel SN, Ayisi J (2007). HIV impairs opsonic phagocytic clearance of pregnancy-associated malaria parasites.. PLoS Med.

[54] Duffy PE, Fried M (2003). Antibodies that inhibit Plasmodium falciparum adhesion to chondroitin sulfate A are associated with increased birth weight and the gestational age of newborns.. Infect Immun.

[55] Dekker GA, Robillard PY, Hulsey TC (1998). Immune maladaptation in the etiology of preeclampsia: a review of corroborative epidemiologic studies.. Obstet Gynecol Surv.

[56] Wolf M, Shah A, Lam C, Martinez A, Smirnakis KV (2005). Circulating levels of the antiangiogenic marker sFLT-1 are increased in first versus second pregnancies.. Am J Obstet Gynecol.

