# Correction: Circulating Soluble Endoglin Levels in Pregnant Women in Cameroon and Malawi—Associations with Placental Malaria and Fetal Growth Restriction

**DOI:** 10.1371/annotation/e9946f72-821d-45ea-bcd4-3fa3ff89a5fe

**Published:** 2011-10-07

**Authors:** Karlee L. Silver, Andrea L. Conroy, Rose G. F. Leke, Robert J. I. Leke, Philomina Gwanmesia, Malcolm E. Molyneux, Diane Wallace Taylor, Stephen J. Rogerson, Kevin C. Kain

The 7th author's correct name is: Diane Wallace Taylor.

The correct author contributions are: Conceived and designed the experiments: KLS ALC RGFL RJIL PG MEM DWT SJR KCK. Performed the experiments: KLS ALC. Analyzed the data: KLS ALC. Contributed reagents/materials/analysis tools: RGFL DWT MEM SJR KCK. Wrote the paper: KLS ALC KCK. Constructive review of manuscript: DWT MEM SJR.

